# Knowledge and disclosure of HIV status among adolescents and young adults attending an adolescent HIV clinic in Accra, Ghana

**DOI:** 10.1186/1756-0500-7-844

**Published:** 2014-11-26

**Authors:** Ernest Kenu, Adjoa Obo-Akwa, Gladys B Nuamah, Anita Brefo, Miriam Sam, Margaret Lartey

**Affiliations:** Korle-Bu Teaching Hospital, Accra, Ghana; Department of Medicine and Therapeutics, University of Ghana Medical School, Accra, Ghana; Department of Epidemiology and Disease Control, School of Public Health, College of Health Sciences, University of Ghana, P.O. Box LG 13, Legon, Ghana

**Keywords:** Knowledge, Disclosure adolescents/young adults, HIV

## Abstract

**Background:**

In Ghana it is estimated that 1.2% of HIV infections occur in young people aged 15-24 but the representation in our clinics is small. Adherence to treatment, appointment keeping and knowledge of HIV status remains a challenge. Disclosure has been shown to result in better adherence to therapy, good clinical outcomes, psychological adjustment and reduction in the risk of HIV transmission when the young person becomes sexually active. A baseline study was conducted to ascertain if adolescents and young adults knew their HIV status and their knowledge on HIV. Informed consent and assent were obtained from willing participants. Self-administered questionnaires on general knowledge of HIV, HIV treatment and disclosure were collected and analyzed.

**Results:**

Thirty-four young persons participated in the study. The mean age was 16.9 ± SD 2.5 and 62% (21/32) were female. All of them were still in school. Eighty-five percent were aware that young people their age could fall sick, 91% had heard of HIV, 70% knew someone with HIV and 45% thought that adolescents were not at risk of HIV. On modes of HIV transmission, 66.7% knew HIV was transmitted through sex and 63.6% knew about mother to child transmission. Fifty three percent (18/34) knew their HIV status, 50% (17/34) were on antiretroviral and 35% (6/17) of them admitted to missing ARV doses. One person who said he was HIV negative and another who did not know his status were both on ARVs.

**Conclusion:**

Disclosure of HIV status to adolescents and young people is dependent on a complex mix of factors and most practitioners recommend an age and developmentally appropriate disclosure. Thus it is highly individualized. The knowledge and awareness of HIV was 91% compared to 97% of adults in the most recent Ghana Demographic and Health Survey however only about two thirds had acceptable in depth knowledge on HIV. Only half knew their HIV status which was not the best considering their ages. There is the need to strengthen education to young persons with HIV, support adhere to ARVs for better outcomes and assist care givers to disclose HIV status to them.

## Background

Children under 15 years of age account for approximately 3.3 million of the people living with human immune virus (HIV)/acquired immune deficiency syndrome (AIDS) worldwide, and almost 90% of all HIV infected children live in sub-Saharan Africa including Ghana [1]. In Ghana the prevalence of HIV in young people from 15-24 years is 1.2% [2]. Currently, children infected with HIV through mother-to-child transmission once diagnosed and in care, live longer and reach adolescence because of greater access to antiretroviral therapy (ART) in the past decade in West Africa [3, 4]. Thus, with the start of national programme for providing care and free highly active antiretroviral therapy (HAART) to children, there has been a significant reduction in morbidity and mortality of HIV infected children and more of them are surviving through childhood into adolescence with improved quality of life [3].

Along with the increased survival of children infected with HIV, disclosure of HIV status to children remains a complex and a critical clinical issue in the care of HIV infected children [4]. World Health organization (WHO) and American Academy of Pediatrics (AAP) strongly encourage disclosing HIV infection status to school aged children and younger children. They should be informed incrementally to accommodate their cognitive skills and emotional maturity [5, 6]. Telling children and adolescents about their HIV infection is a challenging dilemma because children are often asymptomatic in the early stages of HIV infection, yet require daily medications and close monitoring [7].

Caregivers and healthcare workers are presented with group of challenges around disclosure, including deciding on what is in the child’s best interest and when, why and how information about his/her HIV positive status should be shared with him/her. Caregivers are reluctant to disclose the HIV positive status to their children for fear of social rejection and isolation, parental sense of guilt, and fear that the child would not keep diagnosis to themselves [8, 9]. Disclosure of HIV status helps HIV infected adolescents to understand their status and the need for treatment, thereby promoting their participation and responsibility for their care [10]. The rate of disclosure remains low especially in resource limited countries despite the growing evidence of the benefits of disclosure [11]. The inability of most caregivers to handle disclosure has defined the three main patterns of disclosure: complete, partial, and non-disclosure. Complete disclosure of HIV status has been associated with improved adherence to ART [12]. Whereas partial and non-disclosure can strain the relationship between the caregiver and the child. In such situations, force and persuasion are often used to get the child to adhere to treatment; these may results in purposeful rebellion and non-adherence by the child [12].

There is a lot of controversy about what and when to tell children living with HIV about their diagnosis. Many families do not think their children can handle the information and they do not want to upset them because they have already been through so much [8]. Several studies have documented the benefits of disclosure of HIV positive status to HIV infected children and adolescents, including psychological benefits as well as positive effects on the clinical course of the disease [6–9]. The American Academy of Pediatrics recommends that children and adolescents with HIV should be told their diagnosis [6]. However, published rates of disclosure in children from resource rich countries vary widely, from 18 to 77% [6–9, 12], partly due to the lack of conclusive guidelines on when and how to disclose the diagnosis of HIV to children.

Though limited, studies available on rates and effects of HIV disclosure on children are mainly from resource rich countries. There is paucity of data on disclosure from resource limited settings, where the majority of HIV infected children live. Moreover, studies from resource limited settings have mainly been qualitative in design with small samples sizes making it difficult to generalize the findings. This study found out the knowledge and disclosure of HIV status among adolescents and young adults attending adolescent HIV clinic.

## Methods

A cross-sectional study was carried out in July 2013 among HIV infected adolescent and young adults attending an adolescent club meeting in Accra. The Adolescent HIV Care programme at Fevers unit of Korle-Bu Teaching Hospital was established in June 2012 and provides comprehensive HIV/AIDS care and management of opportunistic infections. The Unit serves as the national referral centre for HIV-infected patients. Ever since the first case of HIV was diagnosed in Ghana in 1986, the Unit has provided care and support to persons living with HIV/AIDS. Provision of comprehensive HIV/AIDS care and treatment started at the Unit in December 2003 in line with the scale-up of access to treatment by the National AIDS Control Programme (NACP) and her partners.

Patients are referred from the paediatric HIV clinic to the adult clinic at age twelve. Thus the adult clinic serves both adolescents and adults. An adolescent clinic was started as best practice when it was recognized that their needs and expectations were not being met at the adult clinic. The clinic was supported by adult physicians since it proved difficult to get the paediatricians to come on board.

The adolescents and young adults referred to the clinic present with the following modes of diagnosis: have mothers known to be HIV seropositive during pregnancy through the programme for the prevention of mother-to-child transmission (PMTCT), others are discovered to be infected with HIV after presenting with an AIDS-defining illness, or are diagnosed after either a symptomatic sibling or parent was found to be HIV positive. Majority of them were infected perinatally. At the time of the study, the adolescent clinic was 13 months old and had about 70 HIV infected adolescents and young adults.

Korle-Bu Teaching Hospital gave the approval for the formation of the adolescent group as well as the study. All the young people recruited into the study received medical care for their HIV infection through the clinic and were all invited to voluntarily participate in a general survey on HIV infection at one of the club meetings (Self-selection). Following the self-selection, written informed consent was obtained from those aged 16 years and above and assent and permission from care givers for those less than 16 years after briefing on the purpose, use, and significance of the study. Those who did not consent were excluded from the survey but continued to participate in the fun games, music and dance.

A structured questionnaire was then used to collect data. The questionnaire was anonymous and solicited information on respondents’ background as well as their general knowledge on the causes, mode of transmission of HIV, HIV treatment and disclosure. The questionnaire had three parts. The first part was closed ended and contained questions on the background of the respondents. The second part contained questions on general knowledge and awareness of HIV/AIDS found in HIV/AIDS awareness leaflets used by the Ghana AIDS Commission for information, education and communication. The final part of the questionnaire sought respondents’ views on HIV status, disclosure and treatment. Here, information was collected on disclosure status, age at disclosure, treatment compliance, and socio-demographic data, family type (biologic parents, adoptive/foster, extended), and care- givers HIV status. Service providers and care givers were independently interviewed in a focused group discussion on HIV disclosure. The data collected was entered and analyzed using Statistical Package for the Social Sciences (SPSSÂ®; IBM Corporation, Armonk, NY, USA) version 16 with the key issues being presented in summary frequency tables as descriptive statistics.

## Results

### Demography of respondents

**Figure 1:**
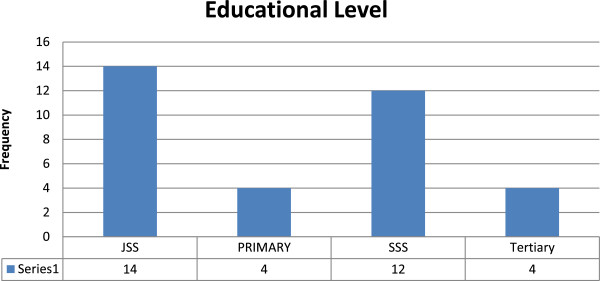
**Educational level of respondents.**

A total of thirty-four (34) young person’s living with HIV from age 13 to 22 were self-recruited to participate in the study out of 36 at the adolescent meeting. Thirty four adolescents and young adults did not attend the adolescent meeting. The mean age of respondents was 16.91â€‰±â€‰2.5, of which 61.8% (21/34) were females. All participants were still in school and the distribution is showed in Figure 1. In relation to religion, 94% (32/34) were Christians and 6% (2/34) were Muslims. The demographic characteristics of the absentees from the meeting were similar to those who attended. Of the 8 care givers in the FGD 7 were females and 1 male whereas 75% (6/8) of the service providers in the FGD were females.

### HIV knowledge

Eighty-five percent (29/34) of respondents were aware that young people of their age could fall sick whilst 5.8% (2/34) thought otherwise and 8.8% (3/34) did not give any response. Over 91% (31/34) of adolescents had heard of HIV as compared to 8.8% (3/34) who had no clue. Moreover, 70.6% (24/34) knew someone with HIV whilst the remaining 29.4% (10/34) did not. Forty-five percent thought that adolescents were not at risk of getting HIV. Fifteen percent (5/34) were not sure whether infected patients could continue with their education. Sub-analysis did not show any statistical difference in HIV knowledge in terms age, sex and educational status.

### Modes of transmission

Majority of the respondents 90.4% (multiple responses) were able to correctly identify one or more mode of HIV of transmission with the remaining 9.6% were not able to provide any correct answers. Out of those who correctly identified the modes of transmission, 66.7% knew that HIV could be transmitted through unprotected sexual intercourse with infected persons and 63.6% knew about mother to child transmission. Twenty three percent (19/83 multiple responses) said HIV can be transmitted by sharing sharp needles/syringes with infected persons. Overall, 29% (10/34) participants think HIV can be transmitted through physical contact.

### The prevalence of HIV disclosure and antiretroviral therapy

**Figure 2:**
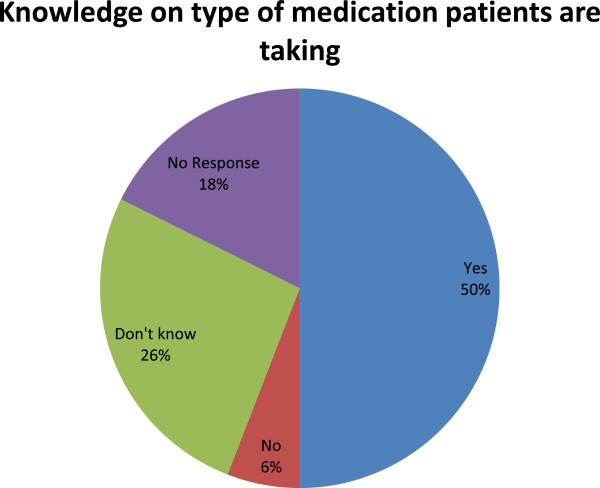
**ART patients’ knowledge on the type of medication they are taking.**

HIV disclosure status rate was 52.9% (18/34), 20.6% (7/34) said they did not have HIV, 20.6% (7/34) did not know whether they had it or not and 5.9% (2/34) did not give any specific answer. Ninety three percent (13/14) of those who did not know their HIV status were between 13 to 15 years. Fifty percent (17/34) of the respondents were on antiretroviral whilst 5.9% (2/34) were not, however 26.5% (9/34) did not know whether they were on ARVs or not. One person who said he was HIV negative but was on ARVs and 17.6% (6/34) did not give any response. In general, 35% (6/17) of the respondents who knew they were on ARVs admitted missing some of their doses Figure 2.

From the FGDs, most of the disclosures happened after ART initiation and were planned. The persons involved in this process varied across individual families. It was sometimes disclosed by family members, particularly care givers or parents after an interview between the latter and a psychologist. None of the respondents did find out their status by chance or through unplanned events.

### Limitation

The study was carried out in a single adolescent clinic with a small sample size which may affect generalizability of our findings.

## Discussion

### Knowledge of HIV

The study revealed that 90.4% were able to correctly identify one or more mode of HIV transmission with the remaining 9.6% not able to provide any correct answer. The fact that 10% of respondents were ignorant on mode of HIV transmission even when they were HIV positive poses serious threats to HIV/AIDS prevention and control in this age group. It was however not clear whether this was just ignorance or denial. This study is in line with findings from other studies done in developing countries such as South Africa and Ethiopia as only about half knew much about their HIV including their status even when on anti-retroviral therapy which was quiet poor [13].

The key to HIV awareness hinges on knowledge of what HIV and AIDS is, and what exposures and risky behaviors can potentially lead to contracting the virus. Knowledge of HIV among infected adolescents and young adults’ is therefore crucial not only in preventing the spread of the virus, but also in addressing the threats posed by HIV/AIDS. There is the need to strengthen education to young persons on HIV as well as support care givers to disclose HIV status and to support young people to adhere to ARVs for better outcomes.

### Status of disclosure

The prevalence of disclosure among our study population was 52.9% (18/34). This is however within the middle bounds of the range (18–77%) reported in studies from resource rich countries. Our finding is consistent but slightly higher than the reported prevalence of disclosure from Thailand, Zambia, and Uganda of 30.1%, 31.8%, and 29%, respectively [6, 7, 12, 14]. Of the children who did not know their HIV positive status, more than two third reportedly were being told of other related diagnosis like TB, malaria, cholera and the rest did not have any clue at all about their illness. Reasons cited by the caregivers for not disclosing HIV status to their wards were consistent with that of studies in resource- limited countries; namely stigmatization and isolation, parental sense of guilt and fear that the child would not keep diagnosis to themselves [10, 15]. As part of standard of care, disclosure is made to adolescents before transfer to the adult clinic. We are unable to tell if these were missed, disclosure was made but not accepted or that adolescents and care givers were still in denial. Interestingly, some caregivers for fear of stigma reverse what healthcare providers say as soon as they leave the presence of healthcare providers. However, several studies from both resource rich and resource limited countries suggest that the disclosure of HIV status to HIV infected adolescents and young adults has immense psychological benefits and positive effects on the clinical course of the disease [5, 12, 16, 17].

Our findings and that of other studies in the sub-region of low rate of disclosure underscore the need for a systematic review of the hurdles to disclosure and implementation of locally and culturally sensitive interventions programs to promote disclosure.

Nearly a third of the caregivers expected the healthcare providers alone to disclose the HIV status of their children. This is contrary to European study where majority of caregivers believed that as parents it was their responsibility to disclose the diagnosis to the child [14]. This may be partly due to the cultural differences in relation to communication between parents and children on issues and the lack of disclosure skills. The healthcare providers agreed that disclosure is the prerogative of the caregivers and children should be told their HIV status. These contrasting views are consistent with that of studies from resource rich countries [14]. The expectation of some caregivers that healthcare providers should disclose the HIV status to the HIV infected adolescents may lead to unnecessary delays in disclosure. As reiterated by the healthcare providers, training in disclosure counseling and a disclosure program adopted by the clinic would allow healthcare providers to better advice caregivers on how to disclose. With caregivers who lack disclosure skills, a counselor assisted or supported disclosure session may suffice [10].

### Transmission of HIV/AIDS

Ninety percent of respondents were able to clearly point out that HIV/AIDS may be transmitted through having unprotected sex with an infected partner, being born to a mother who is infected with HIV, or through the infected mother’s breast milk. Which is similar to the 2008 Ghana Demographic and Health survey (GDHS) report of 97% adult knowledge on HIV transmission [18]. Almost all the adolescents and young adults were also aware that using unsterilized instruments or sharing personal items like toothbrushes could lead to acquiring the virus. Their awareness has a positive impact on prevention of HIV/AIDS considering that sharing items like blades, toothbrushes, pins and needles, cooking and pocket knives, hair and nail clippers, and other sharp objects is a major cause of transmission in less developed countries [16].

The small proportion (10/34) of participants believed that physical contact with infected persons or sharing public space with infected persons could be a source of transmission of HIV/AIDS. This might explain the relatively high stigmatization and discrimination against PLWHA by their colleagues within some communities in Sub Saharan Africa [4]. This is in sharp contrast to the content of educational materials and information provided by the Ghana AIDS Commission, National HIV/AIDS Control Program, and the mass media on HIV/AIDS transmission. These materials clearly explain that it is a scientifically proven fact that physical contact, without the exchange of bodily fluids with an infected person, cannot transmit the virus. In fact, a lot of HIV/AIDS awareness messages in Ghana focus on this point because of the realization that poor awareness of this fact leads to high stigmatization, seclusion, and discrimination of PLWHA. Such perceptions also explain the case made by World Bank that PLWHA in rural areas and less developed countries are sometimes denied access to public transport, swimming in public pools, fetching water from public standpipes, engaging in contact sports, and other activities that involve physical proximity [19].

Believing that physical contact and sharing public space can lead to HIV infection may result in the adolescent and young person self-isolation and self-stigmatization. He or she may not want to socialize and interact for fear of transmitting the virus or becoming infected. These leads to poor social skills, lack of support and subsequently affect adherence and also school work. If such perceptions are still being held by some people, then prevention and even management of new and existing cases of HIV/AIDS may be seriously hindered.

## Conclusions

In conclusion, 47% of HIV infected adolescents in the study were not aware of their HIV status, though strong beneficial effect of HIV disclosure on retention in care after ART initiation beyond the age of ten is known. Disclosure of HIV status to adolescents and young people is dependent on a complex mix of factors and most practitioners recommend an age and developmentally appropriate disclosure thus it is highly individualized. The knowledge and awareness of HIV was 91% compared to 97% of adults in the most recent Ghana demographic and health Survey. There is the need to support care givers to disclose HIV status and to support young people to adhere to ARVs for better outcomes.

### Consent

Written informed consent was obtained from the patient’s guardian/parent/next of kin for the publication of this report and any accompanying images.
